# *De novo* Transcriptome Sequencing Coupled With Co-expression Analysis Reveal the Transcriptional Regulation of Key Genes Involved in the Formation of Active Ingredients in *Peucedanum praeruptorum* Dunn Under Bolting Period

**DOI:** 10.3389/fgene.2021.683037

**Published:** 2021-06-14

**Authors:** Cheng Song, Xiaoli Li, Bin Jia, Li Liu, Jinmei Ou, Bangxing Han

**Affiliations:** ^1^College of Biological and Pharmaceutical Engineering, West Anhui University, Lu’an, China; ^2^Anhui Engineering Laboratory for Conservation and Sustainable Utilization of Traditional Chinese Medicine Resources, West Anhui University, Lu’an, China; ^3^College of Pharmacy, Anhui University of Chinese Medicine, Hefei, China

**Keywords:** *Peucedanum praeruptorum*, transcriptional regulation, coexpression analysis, key gene, coumarins, bolting period

## Abstract

*Peucedanum praeruptorum* Dunn is a perennial and one-off flowering plant of the *Peucedanum* genus in Umbelliferae. The cultivated *P. praeruptorum* Dunn usually grows nutritionally in the first year and then moves into the reproductive growth in the second year. The lignification of the roots caused by bolting leads to the quality decline of crude materials. Since most of the previous studies have dealt with coumarin biosynthesis and identification of functional genes in *P. praeruptorum*, the scientific connotation of the inability that the bolted *P. praeruptorum* cannot be used medically is still unclear. Here, we employed a transcriptome sequencing combined with coexpression analysis to unearth the regulation mechanism of key genes related to coumarin synthesis in pre- and postbolting period, and to explore the mechanisms underlying the effects of bolting on the formation and transport of coumarins between the annual and biennial plants. Six cDNA libraries were constructed, and the transcripts were sequenced and assembled by Illumina Hiseq platform. A total of 336,505 unigenes were obtained from 824,129 non-redundant spliced transcripts. Unigenes (114,488) were annotated to the NCBI nr database, 119,017 and 10,475 unigenes were aligned to Gene Ontology (GO) functional groups and Kyoto Encyclopedia of Genes and Genomes (KEGG) pathways, respectively. Differential expression analysis screened out a series of upregulated and downregulated genes related to the phenylpropanoid pathway. The heatmap clustering showed that the similar expression patterns were both observed in groups C vs. D and groups C vs. F. The WGCNA-based coexpression was performed to elucidate the module and trait relationship to unearth important genes related to the bolting process. Seven pivotal modules on the KEGG functional annotations suggested these genes were mainly enriched in the process of plant–pathogen interaction, plant hormone signal transduction, MAPK signaling pathway, α-linolenic acid metabolism, circadian rhythm, and phenylpropanoid pathway. Further analysis provided clues that the key genes of the phenylpropanoid pathway, the ABC transporters, the apoptosis-related and circadian rhythm regulatory genes may play pivotal roles in regulating bolting signaling, biosynthesis, and transportation of coumarins.

## Introduction

*Peucedanum praeruptorum* Dunn, as a traditional Chinese medicine, is well known for the dried root of the *Peucedanum* genus of the Umbelliferae family. It has multiple effects on dispersing wind and heat, and resolving phlegm (Zhao et al., 2015). Dihydropyran-type coumarin compounds are the main medicinal components of *P. praeruptorum*, including praeruptorin A, praeruptorin B, and praeruptorin C. (Wang et al., 2015). In addition to coumarins, the main chemical components of *P. praeruptorum* also include volatile oils, phenanthrenequinones, organic acids, and sterols (Chu et al., 2020). Studies have proven that the coumarin compounds from *P. praeruptorum* have wide applications in the prevention and treatment of cardiovascular and cerebrovascular diseases, anti-inflammatory, reversing multidrug resistance, anticancer, and neuroprotection (Lee et al., 2015). The methanol extract of the *P. praeruptorum* root could reduce the allergic pneumonia symptoms, and lessen the secretion of mucus and histamine in the airway epithelium, as well as the infiltration of eosinophils (Lee et al., 2017). Some other studies had demonstrated that praeruptorin A could inhibit the migration and invasion of liver cancer cells, and inhibit the expression of the MMP1 gene by activating the extracellular signal-regulated kinase (ERK) signaling pathway, thereby inhibiting the movement of liver cancer cells (Yu et al., 2020). Praeruptorin C had good pharmacological effects in anti-inflammatory, antihypertensive, and antiplatelet aggregation (Liu et al., 2020). Studies had shown that praeruptorin C could significantly inhibit the proliferation, colony formation, wound healing, and migration of the non-small cell lung cancer cells. By inhibiting the phosphorylation of the ERK1/2 signaling pathway, it apparently reduced the expression of cathepsin D and thereby inhibited the invasion activity of the non-small cell lung cancer cells (Liu et al., 2020). In addition, praeruptorin C has a good therapeutic role in improving neuroprotection such as motor and cognitive impairment in Huntington’s disease (Wang et al., 2017).

Recently, the functional genomics has been widely used for the analysis of biosynthetic pathways of specific metabolites of medicinal plants and the mining of functional genes in crucially synthetic pathways (Zhang et al., 2015; Bains et al., 2019; Yu et al., 2019). In particular, the combination of two or more technologies such as genome, transcriptome, proteomics, and metabolomics helps to clarify the formation and molecular regulation mechanism on the TCM efficacy factors (Devi et al., 2016). Coumarin compounds are derived from the phenylpropanoid metabolic pathway. By using high-throughput sequencing and metabonomics technology, some studies had identified some key genes for the synthesis and transport of coumarin compounds, and speculated that the cytochrome P450 family genes and MDR transporters are probably involved in the synthesis and transport of coumarins (Zhao et al., 2015; Song et al., 2020). Phenylalanine ammonia-lyase (*PAL*), as the first rate-limiting enzyme of the phenylalanine pathway, plays an important role in regulating the synthesis of flavonoids, phenols and coumarin compounds. *PpPAL* could respond to abiotic stresses such as jasmonic acid, UV-B, and cold treatment, leading to a rapid increase in the expression level in *P. praeruptorum* (Sui et al., 2019). 4-Coumarate: CoA ligase (4CL) is an important enzyme in the phenylpropanoid-branching pathway, which is responsible for catalyzing the synthesis of cinnamoyl CoA, p-coumaryl CoA, caffeoyl CoA, and ferulic acid CoA. Among the three 4CL genes identified in *P. praeruptorum*, it was found that *Pp4CL1* mainly used coumaric acid and ferulic acid as substrates for the synthesis of downstream metabolites, and could also catalyze precursors such as caffeic acid, cinnamic acid, and o-coumaric acid. However, its paralogous genes *Pp4CL7* and *Pp4CL10* did not show catalytic activity for hydroxycinnamic acid compounds (Liu et al., 2017). The ortho-hydroxylation of hydroxycinnamate is a key step in the synthesis of coumarins and is mainly used for the cyclization of coumarin lactones. p-Coumaroyl CoA 2’-hydroxylase (*C2*’*H*) is a rate-limiting enzyme in the upstream-branching pathway of coumarins, which is used for the synthesis of umbelliferone—the precursor of praeruptorin A. The expression level of *PpC2*’*H* was higher in the roots of *P. praeruptorum*, while the expression was upregulated after methyl jasmonate and UV-B treatments (Yao et al., 2017). The biosynthesis of pectoralum A and B is also required for the participation of some postmodifying enzymes, which are mainly used for methylation, oxy-methylation, prenylation, and redox reactions (Jian et al., 2020). The bergaptol *O*-methyltransferase (*BMT*) participated in the oxygen-methylation reaction of the coumarin bergaptol and had a high substrate specificity (Zhao et al., 2016a,b). Studies had identified a caffeic acid *O*-methyltransferase-similar (*COMT-S*) that from *P. praeruptorum* for the oxygen-methylation reaction of hydroxycoumarin (Zhao et al., 2019b). Further analysis showed that caffeic acid *O*-methyltransferase (*CAOMT*) was obtained from its paralog *BMT* through gene duplication about 37 to 1 million years ago. Due to the increasing need for coumarin compounds in domestic markets, traditional natural extraction can no longer meet the demand. With the rise of synthetic biology, mining specific functional genes will help in the heterologous expression and large-scale production of the medicinal ingredients from *P. praeruptorum*. Using high-throughput sequencing technology, some have identified *PAL*, *4CL*, and *C2*’*H*, three enzymes from *Peucedanum purpurea*, combined with tyrosine ammonia lyase (*TAL*) obtained from *Rhodotorula glutinis*, which removed prephenate dehydratase (*pheA*) and anthranilate synthase (*trpE*), a transcriptional regulatory protein (*tyrR*), to construct a microbial cell production route for the synthesis of the coumarin precursor umbelliferone (Zhao et al., 2019a).

At present, *P. praeruptorum* is still dominated by wild species. With the increase in market demand, wild resources are gradually depleted. The raw materials of *P. praeruptorum* have been unable to meet the needs of the market. As a perennial and one-time flowering plant, the wild *P. praeruptorum* is usually grown for more than 3 years until bolting and flowering. The vegetative growth of *P. praeruptorum* is in the first year of cultivation, and bolting and flowering in the second year (Zhou et al., 2014). Plants such as *Peucedanum*, *Angelica*, *Saposhnikovia*, *Notopterygium*, and *Glehnia* genus of the Umbelliferae family have long been unable to have their roots collected for medicinal use after bolting and flowering. Since the roots of *P. praeruptorum* starts reproductive growth, the roots begin to lignify, and the content of medicinal ingredients is greatly reduced, thus, the cultivated products produced as medicinal materials can only be harvested in the same year (Liang et al., 2018). Early bolting seriously affects the accumulation of secondary metabolites of TCM materials, and has a great impact on the yield and quality of *P. praeruptorum* medicinal materials (Yrjönen et al., 2016). The regulatory mechanism on the inability of the bolted Umbelliferae plants for medicinal use is still unclear. One possible reason is that a large number of nutrients in *P. praeruptorum* are used for bolting in the reproductive growth period, and the roots cannot obtain sufficient nutrition to undertake the normal metabolic activities, which results in increasing the area of secondary xylem and decreasing the content of coumarins (Chen et al., 2019). Another possible reason is that the growth center of *P. praeruptorum* changed upon bolting, and most of the nutrients produced by photosynthesis are transferred from the roots to the apical parts for the development of leaves and floral organs of *P. praeruptorum*. This may be the main reason for the phenomenon of early bolting (Yu et al., 2012). In this study, a high-throughput transcriptome sequencing technology and coexpression analysis was used to analyze the expression patterns of key genes in coumarin biosynthesis before and after bolting, and explored the internal mechanism of bolting on the biosynthesis and transport of coumarins in different *P. praeruptorum*. The experimental samples with different harvest periods, different bolting period and different orientations were collected, and about 330,000 unigenes were identified through transcriptome sequencing. The GO function annotation and KEGG annotation results have shown that pathways such as posttranscriptional modification, signal transduction, and secondary metabolism are highly enriched in these differential genes. Further analysis showed that the key genes of the phenylpropane pathway and ABC transporter, apoptosis-related genes and circadian rhythm regulation genes may both play an important role in regulating bolting, coumarin biosynthesis, and transportation.

## Materials and Methods

### Plant Sample Collection and Pretreatment

The samples used in this experiment were collected from the Donghekou Cultivation Base in Jin’an District, Lu’an City of Anhui Province. The geographical coordinates are 116.6567° east longitude and 31.4044° north latitude. The original plant was identified by Professor Han Bangxing of Wanxi University as *P. praeruptorum* of the *Peucedanum* genus of Umbelliferae. The samples of *P. praeruptorum* were collected between March 2019 and November 2019. First, the enzyme-free tube was precooled with liquid nitrogen. The disinfection and de-RNase treatment of the sampling equipment were performed. Fresh *Peucedanum* root samples were collected, then the surface was quickly cleaned with RNase-free water. The samples were put into an enzyme-free tube for quick freezing in liquid nitrogen. After completely frozen, it was transferred to a refrigerator at −80°C for storage. One hundred milligrams of each *P. praeruptorum* sample was taken and grinded in liquid nitrogen. The RNAprep Pure Plant Kit (Tiangen Biotech (Beijing) Co., Ltd., Beijing, China) was used to extract total RNA from the samples.

### The Transcriptome Sequencing and Assembly

First of all, it is necessary to evaluate and control the quality of raw data obtained. The FastQC method^1^ was used for quality control of raw data. In order to obtain raw reads, the raw data files obtained by the Illumina Hiseq platform are analyzed by base calling and converted to the sequenced reads. The FASTQ file contains sequence read information corresponding with sequencing quality information. The pieces of information, such as the quality value of the original data, were calculated, and FastQC was used to evaluate the quality of the sequencing data of the sample. The Trimmomatic method (Bolger et al., 2014) was used to remove linkers and low-quality sequences in reads to obtain clean data. Trinity (Haas et al., 2013) was used to *de novo* assemble the clean data into a transcript and set the parameter to min_kmer_cov 2. The transcripts assembled by Trinity are non-redundant, and the longest transcript in each transcript cluster is taken as Unigene, which is used as the reference sequence for subsequent analysis. The original data have been uploaded to the SRA database, and the accession of the BioProject was PRJNA714368.

### The Annotations and Gene Structure Analysis

The NCBI blast+ method was used to compare the transcripts with CDD, KOG, COG, NR, NT, PFAM, Swissprot, and TrEMBL databases to obtain functional annotation information (Altschul et al., 1997). According to the annotation of the transcript from Swissprot and TrEMBL, the GO function annotation information is obtained. The KEGG Automatic Annotation Server (KAAS) was used to obtain KEGG annotation information of the transcript (Moriya et al., 2007). By blasting the transcript and the database, the transdecoder software^2^ was used for the CDS prediction. In order to better evaluate the quality of the RNA-seq data, the Bowtie2 software was used to compare the effective data of the samples to the spliced transcripts, and the mapping information was counted (Langmead and Salzberg, 2012). The RSeQC software was used to perform the redundant sequences and insert distribution analysis based on the comparison results (Wang et al., 2012). The BEDTools software was used to make the distribution check and the statistical analysis of gene coverage uniform (Quinlan and Hall, 2010). According to the mapping results, we used the BCFtools to perform the SNP analysis, and filtration was done based on the principle that the quality value is greater than 20, and the coverage is greater than 8 (Li, 2011). The MISA software^3^ was used to perform the SSR analysis based on the sequence information of the spliced transcripts.

### The Analysis of Gene Expression Level

Transcript abundance directly reflects the expression level of a certain gene. In this experiment, we used the TPM value to measure the gene expression level between different treatment groups and Salmon to calculate the gene expression level (Patro et al., 2017). For the repeated samples in the same group, the final expression level is the average of all repeated data. The VennDiagram in the R package was used to construct a common and unique Venn diagram of the expressed genes for all samples. The vegan installation package was used for PCA analysis and PCoA analysis. Also, the vegan package was used to calculate the evolution distance, and then the hclust was used to construct the number of clusters or box plots. The gplots installation package was used to construct a heatmap cluster among samples. The software version and R installation package used in the experiment are shown in Supplementary Table 1.

### The Differential Expression and Co-Expression Analysis

For the samples with biological replicates, DESeq was used for the differential analysis. In order to obtain significantly different genes, we set the selection criteria as: qValue < 0.05 and the multiple of difference| FoldChange| > 2. The scatter plot and volcano plot were used to construct the differentially expressed gene distribution. The VennDiagram was used to construct a Venn diagram of the differential genes. The gplot package was used to construct the heatmap clustering of the differential gene and the expression trend maps of modules. The WGCNA script was to construct a gene set matrix for the correlation analysis of gene coexpression. The differential gene expression profile obtained by transcriptome analysis was used in the WGCNA data set. The soft threshold was further determined by constructing a gene matrix. After selecting a suitable soft threshold, the gene coexpression modules were performed to determine the number of genes in each module. First, the coexpression correlation of coefficient between genes was calculated based on the measured gene expression levels, and then Euclidean distance to cluster the genes by drawing a gene tree was used. The constructed gene tree was pruned by dynamic shearing. The pruned gene tree was fused to obtain gene modules. The differential genes of all groups were selected to visualize the correlation of the genes in the modules by clustering according to the expression amount between genes. A weighted analysis was performed on the phenotypic traits, and the correlation and credibility of all genes in each gene module was calculated with the phenotypic traits. The most relevant and significant modules were chosen as the core module. Finally, the correlation map of module membership and the difference weight of genes were obtained.

### The Enrichment Analysis of the Key Genes

The clusterProfiler in the R package was used for the functional enrichment analysis. The topGO was used to visually analyze GO terms generated by GO enrichment. When the corrected *P*-value is less than 0.05, the function is considered to be significantly enriched. The igraph was used for the correlation analysis of functional enrichment.

## Results and Discussion

### The cDNA Library Construction and Transcript Assembly

**FIGURE 1:**
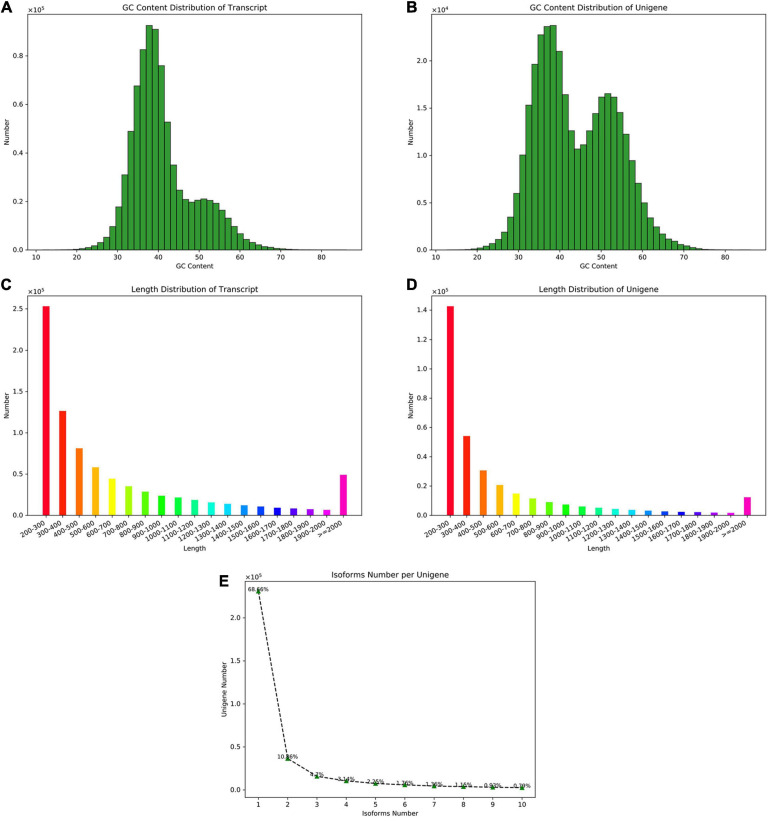
The assembly and sequence length distribution of transcripts and unigenes. **(A)** The GC content distribution of the transcripts. **(B)** The GC content distribution of the unigenes. **(C)** The sequence length distribution of the transcripts. **(D)** The sequence length distribution of unigenes. **(E)** The isoform number of unigenes upon alternative splicing.

The sample information used for sequencing is shown in Supplementary Table 2. A total of 24 samples of *P. praeruptorum* were collected and constructed: A (biennially grown at undrawn phase), B (biennially grown at drawn phase), C (annually grown at undrawn phase), D (annually grown at drawn phase), E (annually grown in north slope), and F (annually grown in south slope) cDNA libraries; each group contains three or six biological replicates. The Illumina Hiseq platform was used to sequence and assemble transcripts of all samples. By removing the linker and the low-quality sequence, the quality control data of all samples is obtained (Supplementary Table 3). The number of spliced transcripts were 824,129, and the number of unigenes were 336,505 (Table 1). By comparing the distribution of GC content and sequence length in the spliced transcript and unigene, it is indicated that the GC content is basically distributed in the range of 40% to 60%, and the number of sequences with a length of 200 to 300 bp is the largest (Figures 1A–D). The variable shear analysis showed that the number of unigenes containing one isoform accounted for 68.6%, and those containing two isoforms accounted for 10.8% (Figure 1E). The results indicated that the obtained unigene could be used for further annotation and the differentially expressed gene analysis.

**TABLE 1:** The detailed information of the spliced transcripts and unigenes.

	**No.**	**≥500 bp**	**≥1,000 bp**	**N50**	**N90**	**Max Len**	**Min Len**	**Total Len**	**Average Len**
Transcript	824129	363,437	173,390	1,082	294	14,871	201	587,521,132	712.9
Unigene	336505	109,099	45,711	799	247	14,871	201	191,946,933	570.41

### The Analysis of the Gene Annotation

**FIGURE 2:**
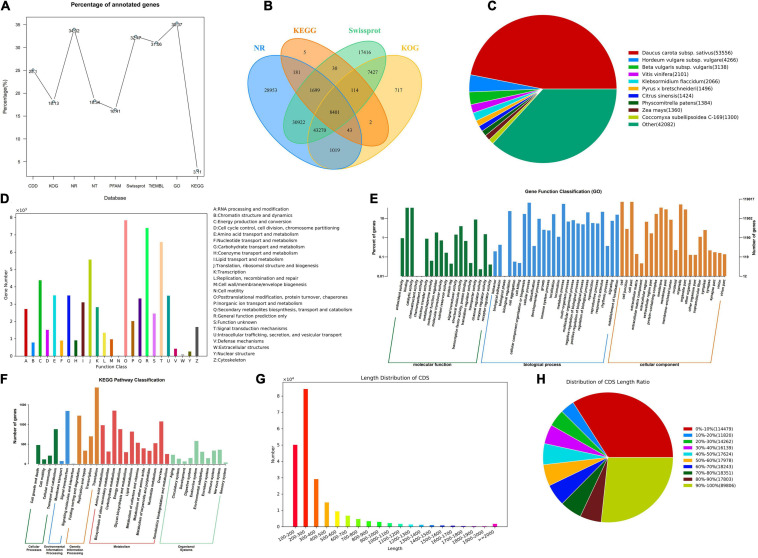
The gene function annotation and the CDS prediction. **(A)** The ratio line chart of the main database annotation. **(B)** The Venn map of the annotated genes based on NR, Kyoto Encyclopedia of Genes and Genomes (KEGG), Swissprot, and KOG. **(C)** The distribution of homologous species based on NR. **(D)** The classification of annotation function based on KOG. **(E)** The Gene Ontology (GO) annotation distribution of different categories. **(F)** The KEGG pathway classification of the annotated genes. **(G)** The length distribution of CDS. **(H)** Distribution of CDS length ratio.

In order to obtain the annotation information of almost all genes, the KOG, COG, NR, NT, PFAM, Swissprot, TrEMBL, and other databases were used for the functional gene annotation. The results indicated that 336,505 genes were annotated under varying databases, of which 114,488 unigenes were annotated to the NR database, and 119,017 were annotated to the GO database, accounting for 34.02% and 35.57% of the total annotated genes, respectively. Swiss-Prot and TrEMBL accounted for 32.47% and 31.06% of the annotated genes, respectively. KEGG had the least functionally annotated genes, accounting for only 3.11% (Figure 2A). The NR, KEGG, Swissprot, and KOG jointly annotated 8,401 genes (Figure 2B). Through the comparison with the NR library, we analyzed the homologous sequence of the transcript of *P. praeruptorum* and its similar species, which found that the transcript of *P. praeruptorum* has the highest similarity with *Daucus carota* subsp. *sativus* (Figure 2C). The annotated genes were classified into 26 groups in KOG. The categories of posttranslational modification, protein turnover, chaperones, signal transduction mechanisms, and general function prediction contained more genes (Figure 2D). The GO function annotations in biological process, cellular component, and molecular function indicated that a majority of the genes were involved in molecular functions such as protein binding, enzyme catalysis, and transport. Some were involved in biological processes such as cell processing, metabolic processes, biological regulation, and environmental induction. A few of the genes were involved in the process, such as composition of cells and organelles (Figure 2E). The classification annotation of KEGG metabolic pathway indicated that more genes were involved in transport and catabolism, signal transduction, folding, sorting, and degradation, transcription, carbohydrate metabolism, amino acid metabolism, and other pathways (Figure 2F). Most CDS lengths are concentrated in 200–300 bp, followed by 100–200 bp (Figure 2G). The higher proportion of the CDS region in the total sequence length is less than 10% or greater than 90% of the sequence (Figure 2H).

### The Gene Structure Analysis

**FIGURE 3:**
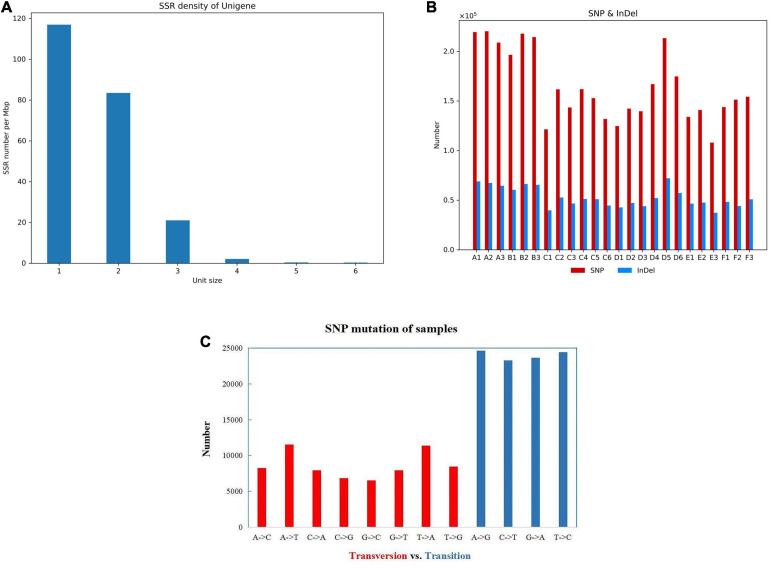
The gene structure and the SNP analysis. **(A)** The density distribution of the SSR marker. The number on the *x*-axis represents the SSR repeat types. **(B)** The amount of SNP and InDel in different groups. **(C)** The SNP mutation of sample A1.

The RSeQC package and BEDTools software were used to analyze the redundant series and gene coverage of the samples. Using MISA performing the SSR detection on the gene transcripts, more than 38,000 SSR markers were identified (Supplementary Table 4). According to the combination and number of bases, it mainly included three types of SSR markers such as single-base repeats, double-base repeats, and three-base repeats (Figure 3A). SNP is a genetic marker formed by a single nucleotide variation, which reflects the polymorphism of a gene. Using BCFtools, SNP/InDel analysis showed that the number of SNP mutant genes in all samples was higher than the number of InDel mutations. SNP mutation sites include two types: transversion and transformation, and the number of transformation mutations is higher than the number of transversion mutation genes (Figures 3B,C).

### The Analysis of Differentially Expressed Genes

**FIGURE 4:**
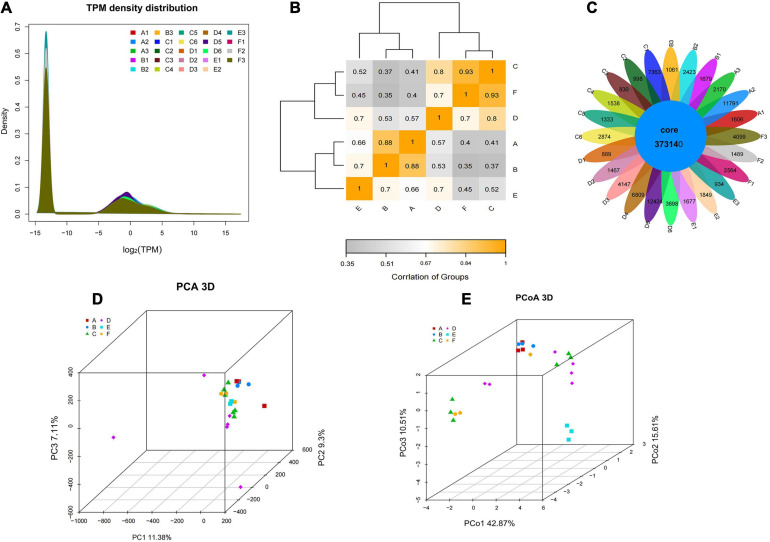
The gene expression level and the intergroup correlation analysis. **(A)** The TPM density distribution between groups. **(B)** Heatmap cluster analysis of the correlation among samples. **(C)** The Venn of coexpression and specific genes. **(D)** PCA visualization of the different groups. **(E)** PCoA visualization of the different groups.

In RNA-seq analysis, the expression level of a gene could be estimated by counting the sequencing sequence (reads) located in the genomic region or gene exon region. In this study, the TPM value was used to measure the expression level of a certain gene (Figure 4A). By comparing the gene expression density curves of the six groups of samples, it was found that the relative density of most non-expressed genes was higher, and the expression interval of some normally expressed genes was set between | log2(TPM)| < 5. The correlation analysis between groups showed that the correlation between group C and group F was higher, as well as group A and group B, which indicated that the gene expression of these samples was similar (Figure 4B). The Venn diagram denoted the number of shared and unique expressed genes (TPM > 0) in the sample. The coexpression Venn showed that 37,314 expressed genes shared in all samples, and some had a certain number of specific expressions (Figure 4C). PCA analysis reflects the distance and aggregation between samples. It was found that the three principal components were unable to separate the six groups (Figure 4D). To observe the differences between individuals and groups, further PCoA analysis showed that group E was separated from other groups, but group A vs. B, group C vs. F, and group C vs. D could not be entirely separated (Figure 4E). It was suggested that these three pairs of groups were collected in the same year (either in the first year or in the second year). Little difference in genetic level was observed in the bolting period, and bolting did not affect the expression level of most genes. The hierarchical cluster analysis and distance analysis between groups also showed that the difference between group A and group B is small, as well as group C vs. D, and group C vs. F. The overall difference between groups is not significant (Supplementary Figure 1).

**FIGURE 5:**
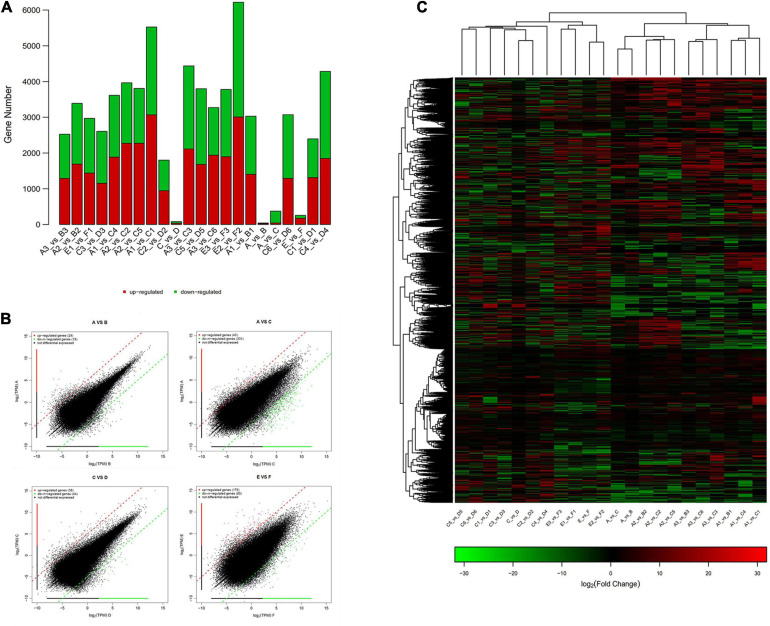
The differential gene expression analysis of *P. praeruptorum*. **(A)** The amount of up- and downregulated DEGs in different groups. **(B)** Scatter plots of the DEGs in A vs. B, A vs. C, C vs. D, and E vs. F. **(C)** Heatmap of the differential genes between groups based on fold change values. The red denotes upregulated expression, and the green denotes downregulated expression.

**FIGURE 6:**
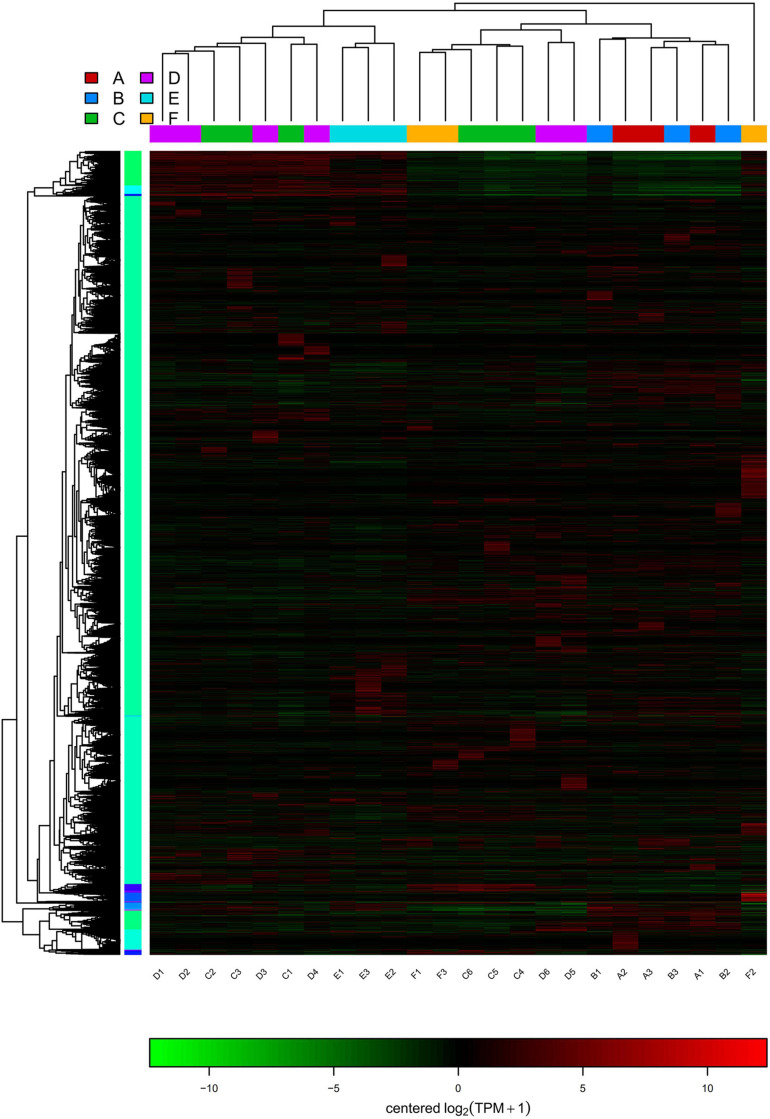
The heatmap clustering of the differential genes between groups.

The statistics of the expressed differential genes showed that the number of differential genes in group A vs. B and group C vs. D was less, and the number of differential genes in group A vs. C and E vs. F was more. Among them, there are 97 and 92 upregulated and downregulated genes in groups A and B, respectively. There are 158 and 225 upregulated and downregulated genes in groups C and D, respectively. There were 685 and 2,248 upregulated and downregulated genes in groups A and C, respectively. There are 2,118 and 1,384 genes that are significantly different between groups E and F (Figure 5A). Figure 5B showed the extremely significant differences between upregulated and downregulated genes (*q*-value < 0.01). The heatmap drawn by fold change indicated that the differential genes of group A vs. B and group A vs. C had similar clustering patterns, and the differential genes of group C vs. D and group E vs. F had similar clusters (Figure 5C). The clustering analysis of DEG clusters showed that the differential genes from groups A and B were clustered into one category, and the differential genes from groups C and D were clustered into one category. However, the distance between two branches of group A and C was relatively long, and group E and F were obviously clustered into two types. The above results suggested that the overall level of gene expression difference of *P. praeruptorum* is relatively small in the bolting phase, and different years and different slope (photoperiod) conditions had a greater contribution to the difference in gene expression (Figure 6). In addition, the expression trend of differential gene clusters showed that the expression levels of most gene sets (subcluster 3 and subcluster 4) tended to be stable, and the expression levels of other gene sets (subcluster 1, subcluster 8, and subcluster 15) appeared uneven pattern (Supplementary Figure 2).

In order to examine the changes in differential gene expression of *P. praeruptorum* before and after bolting, we analyzed the differentially expressed genes of group A vs. B. The results showed that *MAPK1*, *PP2A*, sugar efflux transporter, *DUF4413*, *DUF639*, *PPR*, etc., that related to signal transduction were significantly downregulated after bolting, while *DFR*, *ABCC3*, *ERD6*, protochlorophyllide reductase, seed storage proteins, phloem protein 2 (*PP2*), GDSL lipase, magnesium transporter, and other genes related to growth and development, synthesis, and transportation of secondary metabolites were upregulated (Supplementary Table 5). By comparing the differentially expressed genes of group C vs. D, *ABCB29*, serine hydroxymethyltransferase, *AGL80*, GA-regulated protein, *NRT2.5*, polygalacturonase, *bZIP2*, *bHLH93*, glucose and ribitol dehydrogenase, *CYP81D11*, aquaporin, SBP family genes, anthocyanin acyltransferase, *TCP15*, *RGLG2*, and *WRKY16* were downregulated after bolting. *MYC2*, *ABCC15*, auxin response factor, *ABCC3*, *HCT*, ACC oxidase, carotene 3-hydroxylase, *P5CS*, carotenoid oxygenase, *bZIP35*, *TPS*, *CYP82C3*, *UGT90*, *NAC056*, *NAC043*, *MYB113*, and *NAC025* genes are upregulated after bolting (Supplementary Table 7). The above results indicated that bolting event initiated the transformation of vegetative growth, and the carbon source and some secondary metabolism began to transport from the compound-synthesizing part to the apical parts. The differentially expressed genes of the 1- and 2-year-old unbolted *P. praeruptorum* mainly involved the interaction between plants and pathogenic bacteria, MAPK signaling pathway, hormone signal transduction, starch and sugar metabolism, linolenic acid synthesis, phenylpropane synthesis, and other processes (Supplementary Table 6). *P. praeruptorum* grown in the different directions received different regulation on the photoperiod and intensity. Differential expression analysis showed that starch and sugar metabolism, circadian rhythm, oxidative phosphorylation, hormone signal transduction, carbon fixation in photosynthesis, MAPK signal pathway, fatty acid metabolism, and carbon metabolism are mainly involved in photosynthesis and development processes (Supplementary Table 8).

### Coexpression Analysis and Functional Enrichment of the Differential Gene

**FIGURE 7:**
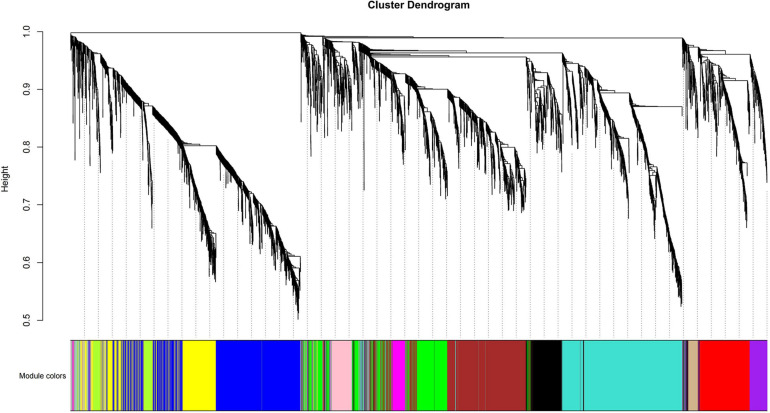
The cluster dendrogram of coexpressed genes.

**FIGURE 8:**
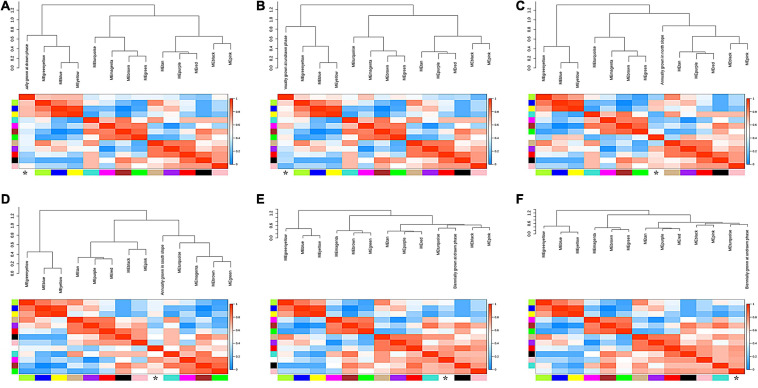
The eigengene adjacency trees of different conditions. **(A)** Annually grown at drawn phase. **(B)** Annually grown at undrawn phase. **(C)** Annually grown in north slope. **(D)** Annually grown in south slope. **(E)** Biennially grown at drawn phase. **(F)** Biennially grown at undrawn phase.

**FIGURE 9:**
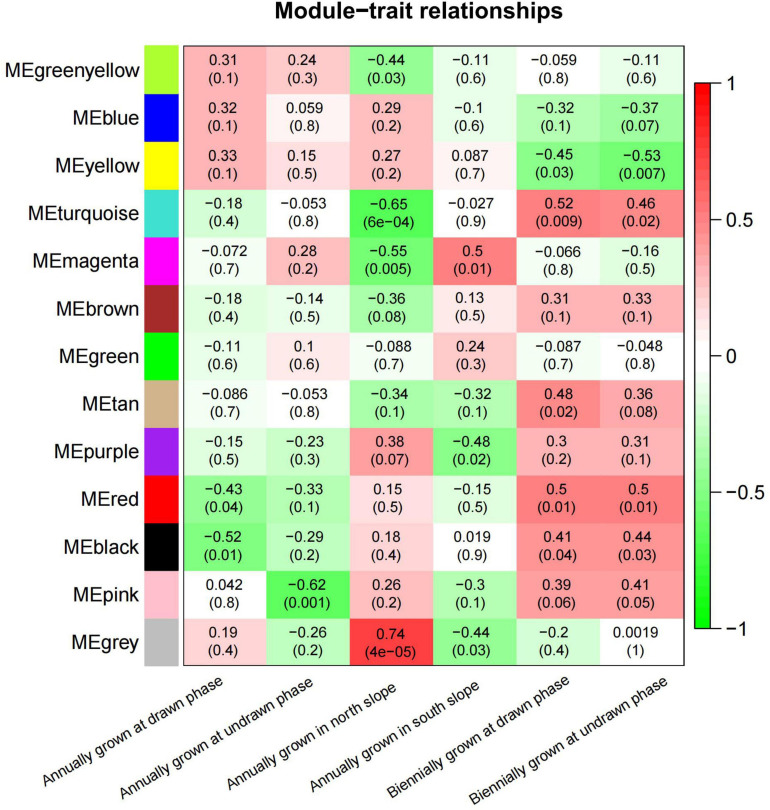
The module–trait relationship between six conditions and modules.

To study the correlation of gene expression, the total expression matrix and all genes were used for the WGCNA analysis. All of 24 samples from six groups were used for the analysis of co-expression. According to the expression profiles of these differential genes, we obtained the WGCNA data matrix to determine the soft threshold. The results showed that if signed R^2 was set to 0.9, the soft threshold was 8, which suggested that it was more appropriate to construct a coexpression matrix under the power value of 8 (Supplementary Figure 3). Euclidean distance was used to cluster genes and draw a cluster dendrogram, which displayed each module through a hierarchical clustering tree. It was indicated that there were 13 gene sets that could form representative modules (Figure 7). By gene coexpression of correlation coefficient, further differentially expressed genes between groups were selected to construct a visualized TOMplot (Supplementary Figure 4). The correlation diagram between these modules obtained by clustering suggested that the module eigengene of different treatment groups were clustered with the modules of close correlation (Figure 8). The asterisks represented the specific position of the six different traits in the modules. For example, the MEgreenyellow module was combined with annually grown at drawn phase and annually grown at undrawn phase. MEpink and MEblack are grouped together with annually grown in the north slope. MEturquiose combined with annually grown in the south slope, biennially grown at drawn and undrawn phase. Moreover, the weighted analysis of traits was performed to obtain the correlation and confidence of all genes and different traits in each module. The results showed that, except for MEgray, MEred and MEgreen were significantly negatively correlated with annually grown at undrawn phase. MEpink had an extremely significantly negative correlation with annually grown at the drawn phase. MEturquoise and MEmagenta are extremely significantly correlated with annually grown in the north slope negatively, while MEturquoise and biennially grown at drawn phase were significantly positively correlated. MEyellow was significantly negatively correlated with biennially grown at undrawn phase (Figure 9). The specific correlation and confidence of module membership and gene significance are shown in Supplementary Figure 5.

**FIGURE 10:**
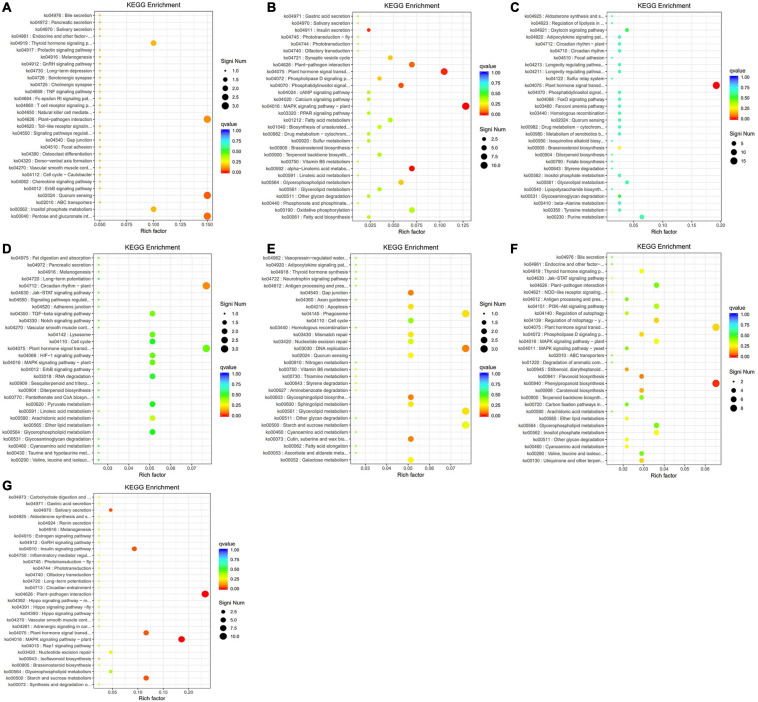
The scatter plots of the significant KEGG enrichment of functional pathway. **(A)** The highly enriched pathways of the black module. **(B)** The highly enriched pathways of the blue module. **(C)** The highly enriched pathways of the brown module. **(D)** The highly enriched pathways of the green module. **(E)** The highly enriched pathways of the red module. **(F)** The highly enriched pathways of the turquoise module. **(G)** The highly enriched pathways of the yellow module. The vertical axis is the function annotation information, and the horizontal axis is rich factor (equal to the number of differential genes annotated to the function divided by the number of genes annotated to the function). *Q* value is shown in different colors, and the number of different genes is shown in the size of dots.

Based on the WGCNA results above, a total of 13 modules with high correlation were obtained. Among them, seven modules (yellow, turquoise, red, green, brown, blue, and black) contain plentifully coexpressed genes. The heatmap clustering matrix indicated that there were more coexpressed genes among blue–yellow, blue–brown, blue–green, blue–blue, brown–yellow, brown–brown, and turquoise–blue modules (Figure 10). Functional enrichment analysis and KEGG pathway analysis could obtain the expression pattern of one typical gene in a specific metabolic pathway. Further GO-enriched annotation on these seven modules indicated that the GO terms annotated in the black module mainly included protein kinase activity, seed development, developmental process involved in reproduction, response to endogenous stimulus and hormone, meristem development, cell wall organization, intrinsic component of membrane, etc. The terms annotated in the black module included serine/threonine kinase activity, kinase activity, response to stress, transferase activity, plasma membrane, oxidoreductase activity, cellulose biosynthetic process, UDP-glucosyltransferase activity, signal transduction, etc. The terms annotated in the Brown module are mainly responses to endogenous stimulus, hormone-mediated signaling pathway, DNA-binding transcription factor activity, etc. The terms annotated in the green module mainly contained transcription regulator activity, circadian rhythm, response to abiotic stimulus, response to oxygen-containing compound, flower development, reproductive shoot system development, etc. The items annotated in the red module mainly included plant-type cell wall, anchored component of plasma membrane, microtubule, polymeric cytoskeletal fiber, hydrolase activity, cytoskeleton, etc. The items annotated in the yellow module mainly included response to endogenous stimulus, response to hormone, DNA-binding transcription factor activity, defense response, response to abiotic and biotic stress, etc. (Supplementary Table 9).

The results of KEGG pathway enrichment analysis are shown in Supplementary Table 10. The enriched genes in the black module included quorum sensing, pentose and glucuronate interversion, plant–pathogen interaction, and signal transduction, translation, carbohydrate metabolism, and other biological processes (Figure 10A). The enrichment in the blue module contained MAPK signaling pathway, plant hormone signal transduction, α-linolenic acid metabolism, transport, and catabolism, and lipid metabolism (Figure 10B). The enriched genes in the brown module are mainly involved in plant hormone signal transduction (Figure 10C). The enriched genes in the green module are mainly involved in the circadian rhythm (Figure 10D). The red module is mainly involved in DNA replication, glycerolipid metabolism, and starch and sucrose metabolism (Figure 10E). The enriched genes in the turquoise module are mainly involved in the phenylpropanoid biosynthesis and plant hormone signal transduction (Figure 10F). The yellow module is mainly involved in plant-pathogen interaction, MAPK signaling pathway, starch and sucrose metabolism, plant hormone signal transduction, and environmental adaptation (Figure 10G). Finally, the differential genes from the seven modules based on KEGG enrichment are described in Table 2.

**TABLE 2:** The significantly differential genes of the Kyoto Encyclopedia of Genes and Genomes (KEGG) enrichment in seven modules.

**Modules**	**Unigene ID**	**Description**	**KEGG term**
Black	TRINITY_DN91982_c0_g1,TRINITY_DN97897_c1_g1,TRINITY_DN94663_c1_g5	Pectate lyase, pectate lyase, nonspecific phospholipase	Quorum sensing
	TRINITY_DN91982_c0_g1,TRINITY_DN97897_c1_g1,TRINITY_DN80952_c0_g1	Pectate lyase, pectate lyase, pectinesterase	Pentose and glucuronate interconversions
	TRINITY_DN92836_c0_g1,TRINITY_DN74599_c2_g1,TRINITY_DN78227_c0_g4	CDK30, MKK6, EDS1	Plant–pathogen interaction
Blue	TRINITY_DN69364_c0_g2,TRINITY_DN93921_c0_g2,TRINITY_DN93921_c0_g1, TRINITY_DN87065_c0_g1,TRINITY_DN78780_c1_g1,TRINITY_DN77043_c0_g3, TRINITY_DN90039_c0_g1,TRINITY_DN69537_c0_g1,TRINITY_DN80918_c1_g1, TRINITY_DN96204_c0_g1,TRINITY_DN70380_c1_g4	MKS1, serine/threonine–protein kinase OXI1, OXI1, bHLH14, PP2Cc, MYC2, MKKA, MKKK4, WRKY22, MPK9, MKS1	MAPK signaling pathway—plant
	TRINITY_DN98338_c2_g1,TRINITY_DN91314_c0_g2,TRINITY_DN91314_c0_g1, TRINITY_DN88300_c0_g1,TRINITY_DN98156_c2_g2,TRINITY_DN93109_c0_g3	LOX2, AOC4, AOC4, OPR2, AOC3, ACX1	Alpha-linolenic acid metabolism
	TRINITY_DN82132_c0_g1,TRINITY_DN90894_c0_g2,TRINITY_DN73073_c1_g1, TRINITY_DN87479_c2_g5,TRINITY_DN87065_c0_g1,TRINITY_DN78780_c1_g1, TRINITY_DN77043_c0_g3,TRINITY_DN84473_c4_g1,TRINITY_DN94676_c0_g3	TIFY10A, XTH23, TIFY10A, TIFY10A, bHLH14, PP2Cc, MYC2, GID1B, BZR1	Plant hormone signal transduction
	TRINITY_DN83723_c0_g2,TRINITY_DN92948_c0_g1,TRINITY_DN95105_c1_g1, TRINITY_DN98530_c3_g1,TRINITY_DN93972_c0_g1	CML45, PLC4, DGK1, DGK5, DGK2	Phosphatidylinositol signaling system
	TRINITY_DN79382_c1_g1,TRINITY_DN83723_c0_g2,TRINITY_DN69924_c1_g1, TRINITY_DN66938_c0_g1,TRINITY_DN80918_c1_g1,TRINITY_DN96169_c1_g1	Calcium-binding allergen 8, CML45, CPK7, CML23, WRKY22, respiratory burst oxidase homolog protein C	Plant–pathogen interaction
Brown	TRINITY_DN86120_c2_g3,TRINITY_DN72157_c2_g1,TRINITY_DN89061_c0_g2, TRINITY_DN79989_c1_g2,TRINITY_DN73550_c1_g1,TRINITY_DN88273_c1_g1, TRINITY_DN89338_c2_g1,TRINITY_DN87704_c0_g2,TRINITY_DN75691_c0_g1, TRINITY_DN81580_c1_g1,TRINITY_DN91987_c1_g4,TRINITY_DN76934_c0_g1, TRINITY_DN80774_c0_g1,TRINITY_DN83614_c0_g1,TRINITY_DN87173_c0_g1	SAUR72, ARF18, PP2Cc, TGA-2.1, SAUR32, bZIP7, ARR9, IAA13, ABF2, IAA11, PYL9, bZIP8, GBF4, ARR3, DELLA protein GAI	Plant hormone signal transduction
	TRINITY_DN95742_c1_g1,TRINITY_DN98250_c0_g1	CYP90D1, CYP749A22	Brassinosteroid biosynthesis
	TRINITY_DN79675_c0_g3,TRINITY_DN79269_c0_g2	Heparan-α-glucosaminide *N*-acetyltransferase, heparanase-like protein 3	Glycosaminoglycan degradation
	TRINITY_DN98033_c2_g1,TRINITY_DN94990_c1_g3	GIGANTEA, TCP7	Circadian rhythm—plant
Green	TRINITY_DN96996_c3_g1,TRINITY_DN89650_c0_g2,TRINITY_DN78434_c1_g1	CRY1, APRR5, CCA1	Circadian rhythm—plant
	TRINITY_DN92859_c0_g1,TRINITY_DN94016_c0_g3	GGT1, phospholipase A2-alpha	Arachidonic acid metabolism
	TRINITY_DN94016_c0_g3	Phospholipase A2-alpha	Linoleic acid metabolism
Red	TRINITY_DN93413_c1_g1,TRINITY_DN93413_c0_g1	Alpha-galactosidase, alpha-galactosidase	Glycosphingolipid biosynthesis
	TRINITY_DN92960_c0_g1,TRINITY_DN71353_c4_g2	Fatty acid omega-hydroxylase, fatty aldehyde decarbonylase	Cutin, suberin, and wax biosynthesis
	TRINITY_DN87965_c0_g2,TRINITY_DN93413_c1_g1,TRINITY_DN93413_c0_g1	PDAT2, alpha-galactosidase, alpha-galactosidase	Glycerolipid metabolism
Turquoise	TRINITY_DN77989_c3_g2,TRINITY_DN86350_c0_g1,TRINITY_DN75021_c0_g3, TRINITY_DN95188_c0_g1,TRINITY_DN95807_c3_g1,TRINITY_DN81440_c1_g1, TRINITY_DN95494_c1_g1,TRINITY_DN74684_c0_g2,TRINITY_DN81857_c0_g3	CCoAOMT, PER21, *O*-acetyltransferase, CAOMT, beta-glucosidase, CAOMT, C4H, PER51, CCR1	Phenylpropanoid biosynthesis
	TRINITY_DN77989_c3_g2,TRINITY_DN75021_c0_g3,TRINITY_DN96835_c0_g1, TRINITY_DN95494_c1_g1	CCoAOMT, *O*-acetyltransferase, chalcone isomerase, C4H	Flavonoid biosynthesis
	TRINITY_DN77989_c3_g2,TRINITY_DN75021_c0_g3,TRINITY_DN95494_c1_g1	CCoAOMT, *O*-acetyltransferase, C4H	Stilbenoid, diarylheptanoid and gingerol biosynthesis
	TRINITY_DN92852_c0_g3,TRINITY_DN74872_c1_g1,TRINITY_DN70885_c1_g1, TRINITY_DN89214_c0_g1	NCED1, CCD8B, CYP97A3, VDE	Carotenoid biosynthesis
Yellow	TRINITY_DN75582_c1_g3,TRINITY_DN87724_c1_g3,TRINITY_DN83018_c0_g4, TRINITY_DN95142_c1_g2,TRINITY_DN88604_c0_g1,TRINITY_DN88820_c1_g3, TRINITY_DN85142_c0_g4,TRINITY_DN76663_c0_g1, TRINITY_DN76663_c0_g3,TRINITY_DN69751_c0_g2	CML19, WRKY33, CML25, WRKY33, WRKY33, WRKY22, CML27, RIN4, RIN4, CML45	Plant–pathogen interaction
	TRINITY_DN87724_c1_g3,TRINITY_DN95142_c1_g2,TRINITY_DN88604_c0_g1, TRINITY_DN86043_c0_g1,TRINITY_DN88820_c1_g3,TRINITY_DN93901_c2_g1, TRINITY_DN73035_c0_g1,TRINITY_DN86457_c0_g1	WRKY33, WRKY33, WRKY33, ABCB22, WRKY22, PYL4, PYL5, MKK9	MAPK signaling pathway—plant
	TRINITY_DN88900_c1_g4,TRINITY_DN98074_c1_g1,TRINITY_DN95611_c1_g2, TRINITY_DN89504_c0_g1,TRINITY_DN79580_c1_g1	*O*-Glycosyl hydrolase, alpha- amylase 2, beta-amylase 3, UDP-glucuronate 4-epimerase, beta-amylase 3	Starch and sucrose metabolism

**FIGURE 11:**
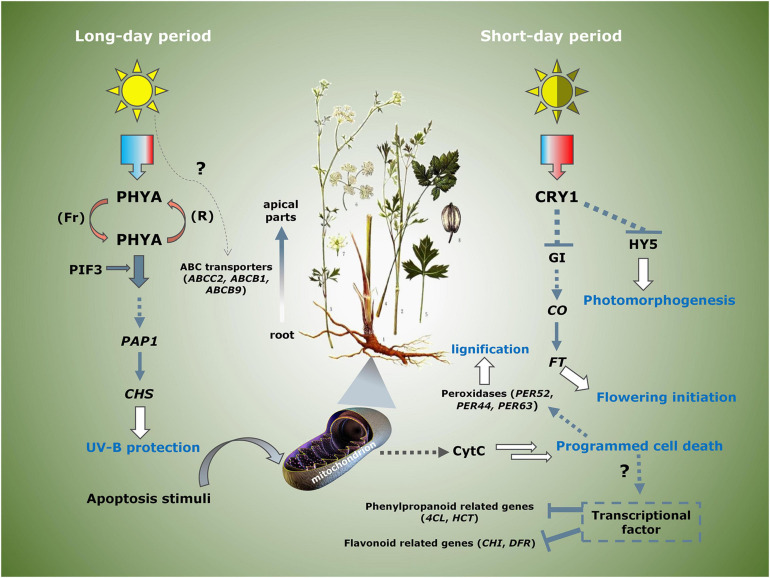
A plausible model on the molecular mechanism of photoperiod regulating bolting and transformation of coumarins in *P. praeruptorum*. *PHYA* (TRINITY_DN77328_c0_g1), *PIF3* (TRINITY_DN83313_c0_g2), *PAP1* (TRINITY_DN63777_c0_g1, TRINITY_DN63757_c0_g2), *CRY1* (TRINITY_DN82168_c0_g4, TRINITY_DN96996_c3_g1), *GI* (TRINITY_DN87544_c1_g2), *CO* (TRINITY_DN75433_c0_g3, TRINITY_DN94065_c2_g1), *FT* (TRINITY_DN110183_c0_g1, TRINITY_DN61953_c0_g1), *HY5* (TRINITY_DN89278_c0_g1, TRINITY_DN5919_c0_g1), *CHS* (TRINITY_DN70261_c1_g1, TRINITY_DN91517_c0_g1, TRINITY_DN80066_c0_g1), *CytC* (TRINITY_DN69779_c1_g2, TRINITY_DN70114_c2_g1).

To explore the key genes that regulated the phenylpropane pathway further, we performed KEGG enrichment analysis on the differential genes between the groups (Table 3). By comparing the differentially expressed genes of group A vs. C, it indicated that most of the peroxidases related to the formation of lignin monomers were apparently downregulated, and the *CHS* related to flavonoid biosynthesis was upregulated. *4CL* and *HCT*, which are involved in the synthesis of phenylpropanoid, were downregulated, while the ABC transporters involved in coumarin transport were upregulated. Group C vs. D involved the differentially expressed genes on bolting. It was indicated that phosphatidylinositol signaling and apoptosis genes were the main differential genes, suggesting that they may be involved in the process of programmed cell necrosis (PCD) of root cells. Compared with group F, the genes involved in the synthesis and transport of phenylpropanoids and flavonoids of group E were almost downregulated, indicating that long-day period was beneficial to induce the expression of these genes. Interestingly, we have also identified several genes related to the circadian rhythm. Phytochrome A was upregulated in short-day period, and *CRY* and *GI* genes are upregulated in long-day period, indicating that the two types of genes that jointly regulated the photoperiod flowering and development of *P. praeruptorum* are in different proportions of red and blue light. In summary, we proposed a photoperiod-dependent working model for the growth and regulation of secondary metabolites of *P. praeruptorum* (Figure 11). The photoperiod and apoptosis signaling jointly participated in the regulation of the bolting period and later growth. Under long-day conditions, PHYA and PIF3 were coordinated to regulate the expression of downstream CHS related to UV-B light stress. Under short-day conditions, CRY participated in the expression of CONSTANS, which, in turn, enhanced the upregulated expression of FT to initiate flowering, followed by regulating HY5 to control its photomorphogenesis. The apoptosis process mainly involved the programmed death of root cells, which further affected the lignification process and the expression levels of related biosynthesis genes. This study will provide a scientific reference for the bolting and flowering mechanism of *P. praeruptorum* and the regulation of key genes in coumarin biosynthesis.

**TABLE 3:** The significantly different genes between groups.

**Groups**	**Unigene ID**	**Description**	**Log_2_(fold change)**	**KEGG term**
A vs. C	TRINITY_DN78443_c0_g1	26-*O*-β-glucosidase	–1.69	Phenylpropanoid biosynthesis
	TRINITY_DN90447_c0_g1	Cationic peroxidase 2	–4.51	
	TRINITY_DN88245_c0_g2	PER64	6.33	
	TRINITY_DN88245_c0_g3	PER64	7.17	
	TRINITY_DN88958_c1_g6	β-Glucosidase 12	–1.08	
	TRINITY_DN78489_c3_g1	Glutathione peroxidase	–4.16	
	TRINITY_DN88179_c1_g2	HCT	–3.87	
	TRINITY_DN79471_c0_g4	HCT	–10.53	
	TRINITY_DN92248_c0_g1	PER44	–10.71	
	TRINITY_DN92351_c2_g2	4CL	–1.38	
	TRINITY_DN79471_c0_g4	HCT	–10.54	
	TRINITY_DN95807_c3_g1	β-Glucosidase 11	2.66	
	TRINITY_DN74096_c0_g1	ABCB1	3.02	ABC transporters
	TRINITY_DN96811_c2_g1	ABCB9	2.68	
	TRINITY_DN74096_c1_g1	ABCB1	3.36	
	TRINITY_DN88179_c1_g2	HCT	–3.87	Flavonoid biosynthesis
	TRINITY_DN91517_c0_g1	CHS1	3.86	
	TRINITY_DN79471_c0_g4	HCT	–10.53	
C vs. D	TRINITY_DN88080_c0_g1	β-Glucosidase 44	–3.70	Phenylpropanoid biosynthesis
	TRINITY_DN93179_c0_g3	IPK2b	6.14	Phosphatidylinositol signaling system
	TRINITY_DN68773_c0_g2	IPK2a	4.47	
	TRINITY_DN86197_c2_g3	PLC2	4.89	
	TRINITY_DN86197_c2_g1	PLC2	4.83	
	TRINITY_DN69779_c1_g2	Cytochrome c	–4.95	Apoptosis—multiple species
	TRINITY_DN70114_c2_g1	Cytochrome c	–3.34	
E vs. F	TRINITY_DN78443_c0_g1	β-Glucosidase	–3.01	Phenylpropanoid biosynthesis
	TRINITY_DN93468_c0_g1	PER63	1.88	
	TRINITY_DN91057_c0_g1	4CL	–3.63	
	TRINITY_DN69279_c0_g1	PER52	–4.59	
	TRINITY_DN92351_c2_g2	4CL	–3.83	
	TRINITY_DN97609_c1_g1	DFR	–2.96	Flavonoid biosynthesis
	TRINITY_DN80821_c0_g2	CHI	–7.89	
	TRINITY_DN92803_c1_g2	ABCC2	–2.69	ABC transporters
	TRINITY_DN95608_c1_g1	ABCC2	–3.98	
	TRINITY_DN82168_c0_g4	CRY	–3.10	Circadian rhythm—plant
	TRINITY_DN87544_c1_g2	GI	–4.72	
	TRINITY_DN96996_c3_g1	CRY	–4.12	
	TRINITY_DN77328_c0_g1	PHYA	2.71	

## Conclusion

Bolting is often the turning point of plants from vegetative growth to reproductive growth. However, the scientific connotation for which the crude material of bolted *P. praeruptorum*, hardly used for medicinal purposes, need to be addressed. By transcriptome sequencing technology, the gene expression profiles of *P. praeruptorum* was employed under different conditions, by which a series of key genes related to phenylpropanoid metabolism and transportation was filtered. Differential gene analysis and coexpression further revealed the connection between growth and secondary metabolism. We inferred that the physiological bolting may not be the direct cause of the downregulation of genes involved in the coumarin synthesis pathway. The programmed cell death and the photoperiod regulation could probably be the reason for leading the lignification of roots and caused the chemical components to migrate to the apical parts after bolting. Some evidence proved that numerous materials were supplemented in the reproductive growth period. The underground part like taproot may not obtain sufficient nutrition to undertake the normal metabolism, which leads to thickening of the secondary xylem and the reduction of coumarins. The results will provide theoretical support for the early flowering intervention, coumarin biosynthesis, and transport of *P. praeruptorum*.

## Data Availability Statement

The datasets presented in this study can be found in online repositories. The names of the repository/repositories and accession number(s) can be found in the article/Supplementary Material.

## Author Contributions

BH, CS, and XL designed the research. CS, XL, BJ, and LL conducted the experiments. CS, XL, and JO analyzed the data. CS wrote the manuscript. BH, CS, JO, and XL revised the manuscript and improved the English. BH and CS acquired the funding. All authors have read, reviewed, and approved the submitted version.

## Conflict of Interest

The authors declare that the research was conducted in the absence of any commercial or financial relationships that could be construed as a potential conflict of interest. The handling editor declared a past collaboration with one of the authors CS.

## Footnotes

1 http://www.bioinformatics.babraham.ac.uk/projects/fastqc/

2 http://transdecoder.github.io/

3 https://webblast.ipk-gatersleben.de/misa/

## Supplementary Material

Supplementary Figure 1 The distance similarity analysis between samples. **(A)** Hierarchical clustering tree. **(B)** Heatmap of distance between samples. **(C)** Analysis of anosim group similarity. If *R*-value is close to 1, difference between groups become greater. If *R*-value is close to 0, suggesting there is no significant difference between and within groups. The *p*-value denote the significance of this statistical analysis.

Supplementary Figure 2 The expression trend of different gene modules.

Supplementary Figure 3 The soft threshold plot of scale free topology model. The dot line denoted the signed *R*^2^ value at 0.9.

Supplementary Figure 4 The TOM plot with cluster dendrogram. The yellow matrix across diagonal represents the correlation between modules, and the density represents the number of co-expressed genes.

Supplementary Figure 5 The module membership of different conditions with gene significant weight. **(A)** Annually grown at drawn phase. **(B)** Annually grown at undrawn phase. **(C)** Annually grown in north slope. **(D)** Annually grown in south slope. **(E)** Biennially grown at drawn phase. **(F)** Biennially grown at undrawn phase.

Supplementary Table 1 The software version and R packages used in the experiment.

Supplementary Table 2 The detailed information of the samples in the experiment.

Supplementary Table 3 The QC data statistics of all samples.

Supplementary Table 4 The SSR markers of unigenes.

Supplementary Table 5 The significantly differential expressed genes between group A and B.

Supplementary Table 6 The significantly differential expressed genes between group A and C.

Supplementary Table 7 The significantly differential expressed genes between group C and D.

Supplementary Table 8 The significantly differential expressed genes between group E and F.

Supplementary Table 9 The functional GO enrichment of seven modules obtained by co-expression analysis.

Supplementary Table 10 The KEGG enrichment of seven modules obtained by co-expression analysis.
