# Supplementary figures and images for: De novo Transcriptome Sequencing Coupled With Co-expression Analysis Reveal the Transcriptional Regulation of Key Genes Involved in the Formation of Active Ingredients in Peucedanum praeruptorum Dunn Under Bolting Period
Source: Front Genet. 2021 Jun 14;12:683037. doi: 10.3389/fgene.2021.683037 (PMC8236723; doi:10.3389/fgene.2021.683037)

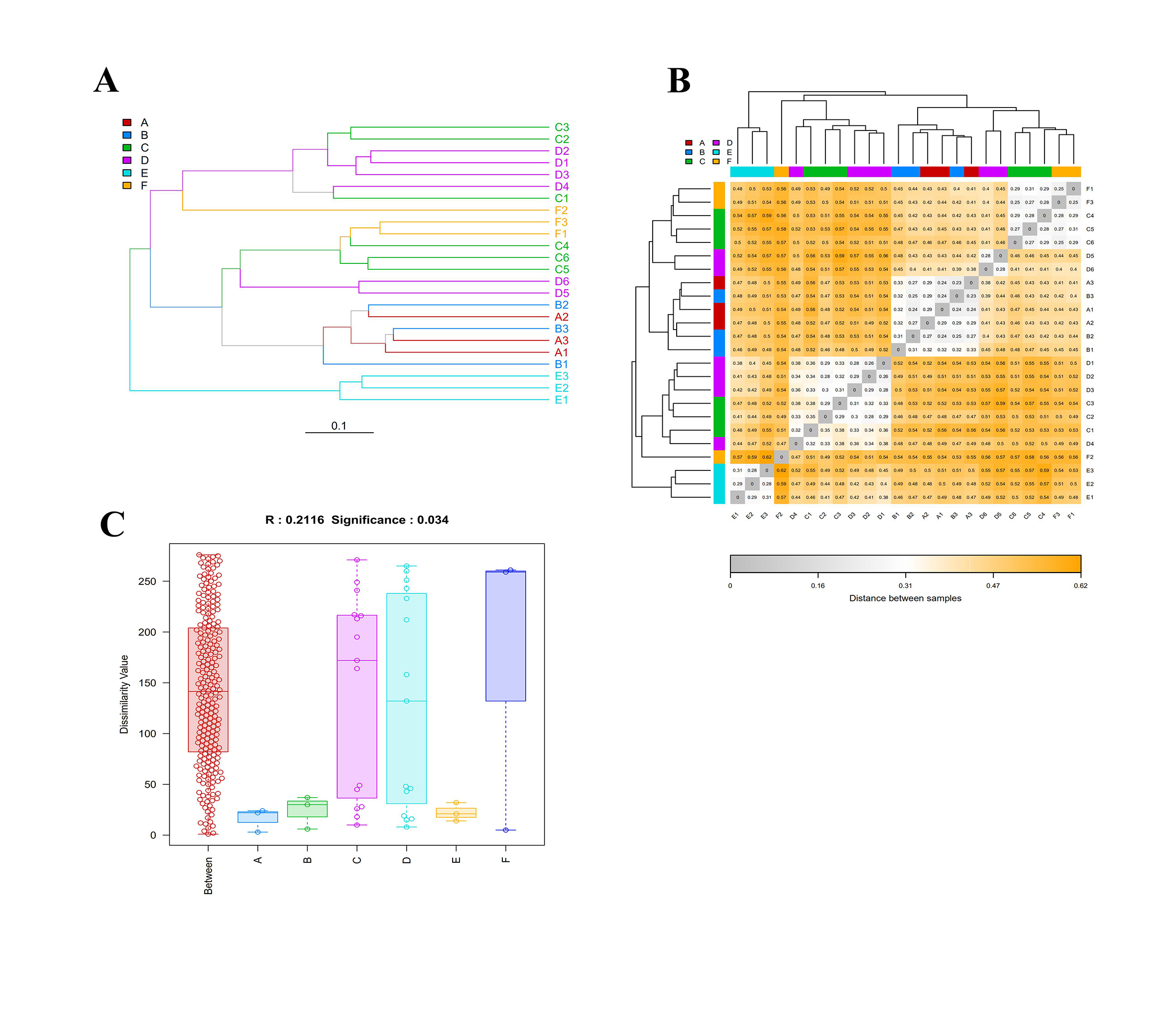

Supplement: Supplementary Figure 1 — The distance similarity analysis between samples. (A) Hierarchical clustering tree. (B) Heatmap of distance between samples. (C) Analysis of anosim group similarity. If R-value is close to 1, difference between groups become greater. If R-value is close to 0, suggesting there is no significant difference between and within groups. The p-value denote the significance of this statistical analysis. [file Image_1.JPEG]

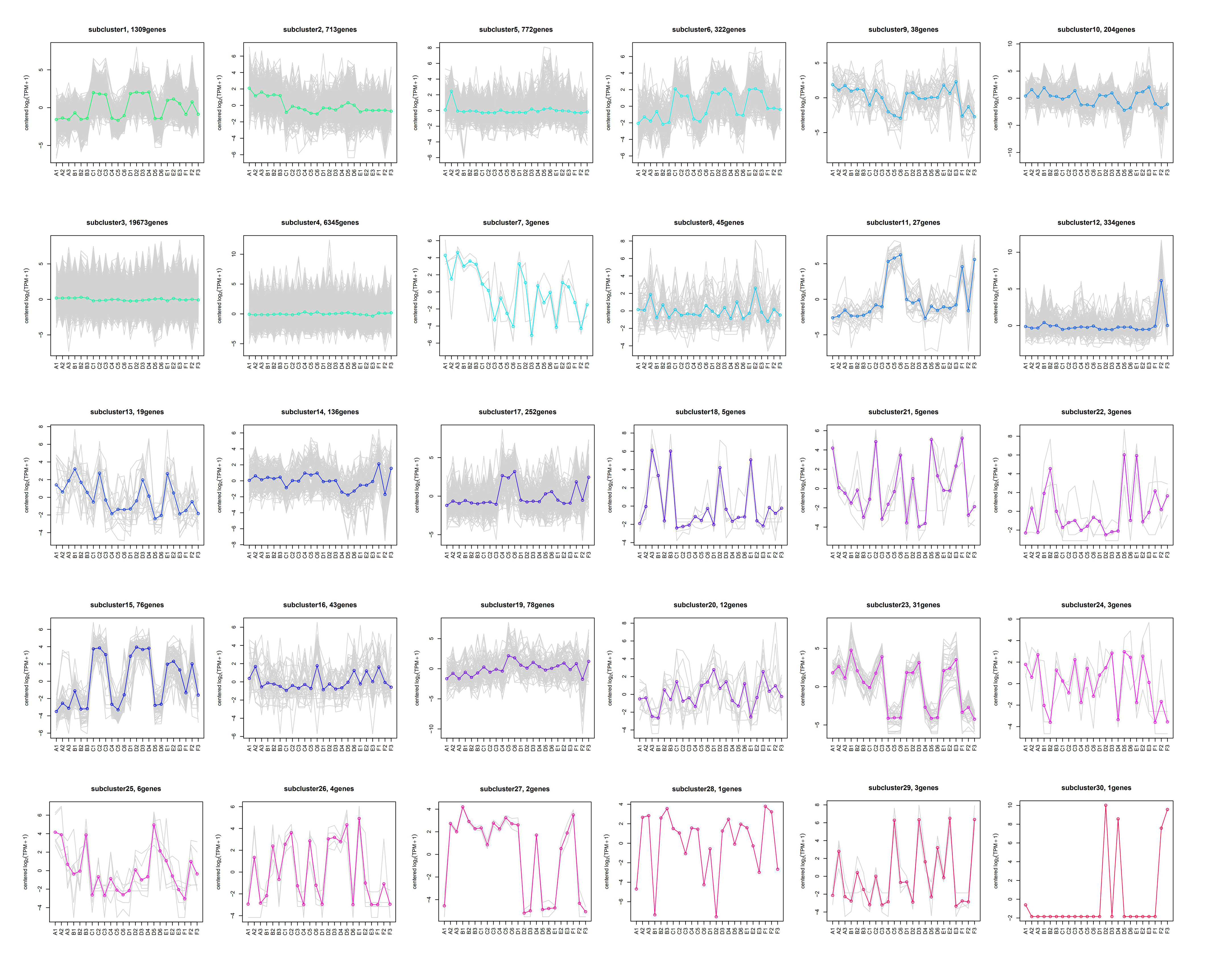

Supplement: Supplementary Figure 2 — The expression trend of different gene modules. [file Image_2.JPEG]

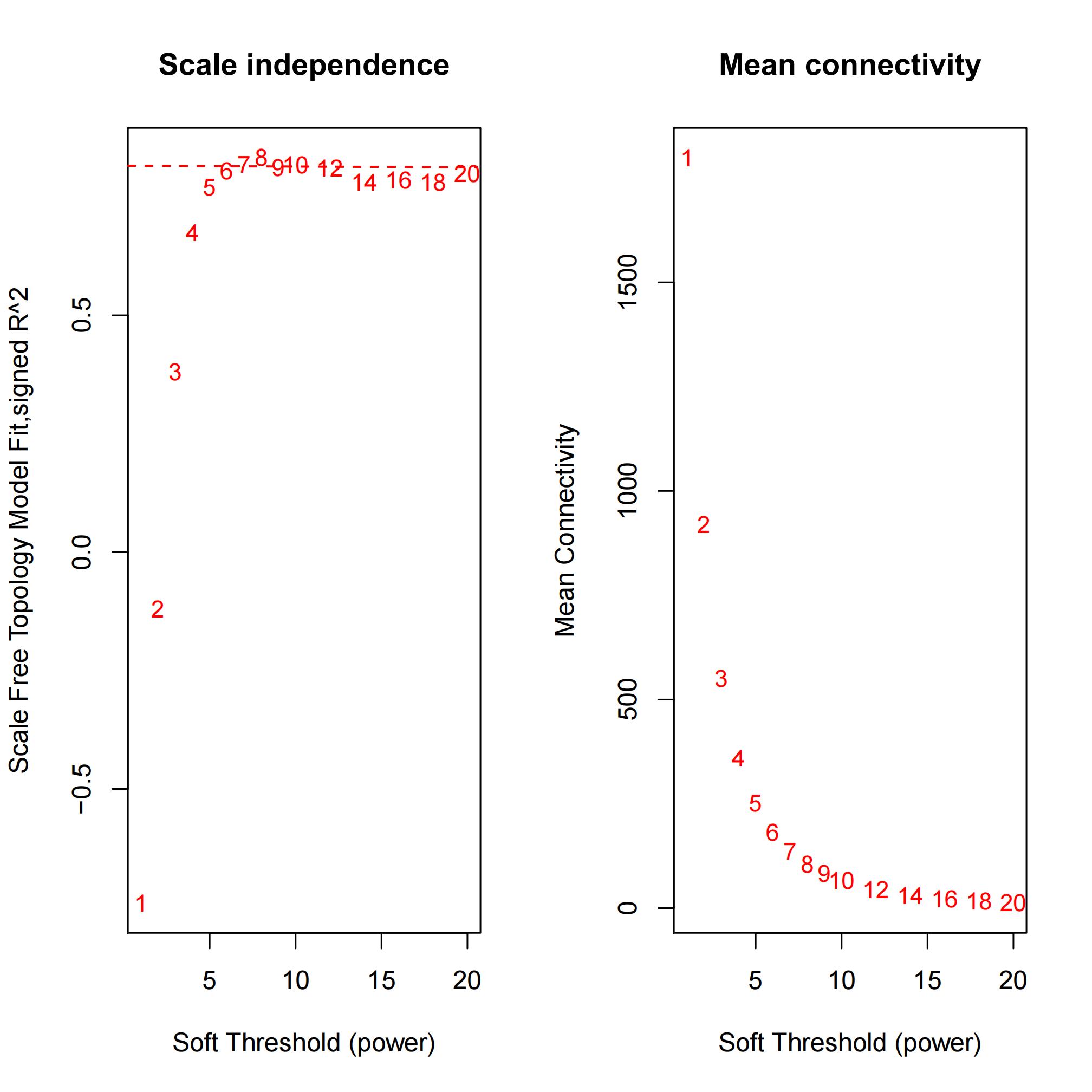

Supplement: Supplementary Figure 3 — The soft threshold plot of scale free topology model. The dot line denoted the signed R2 value at 0.9. [file Image_3.JPEG]

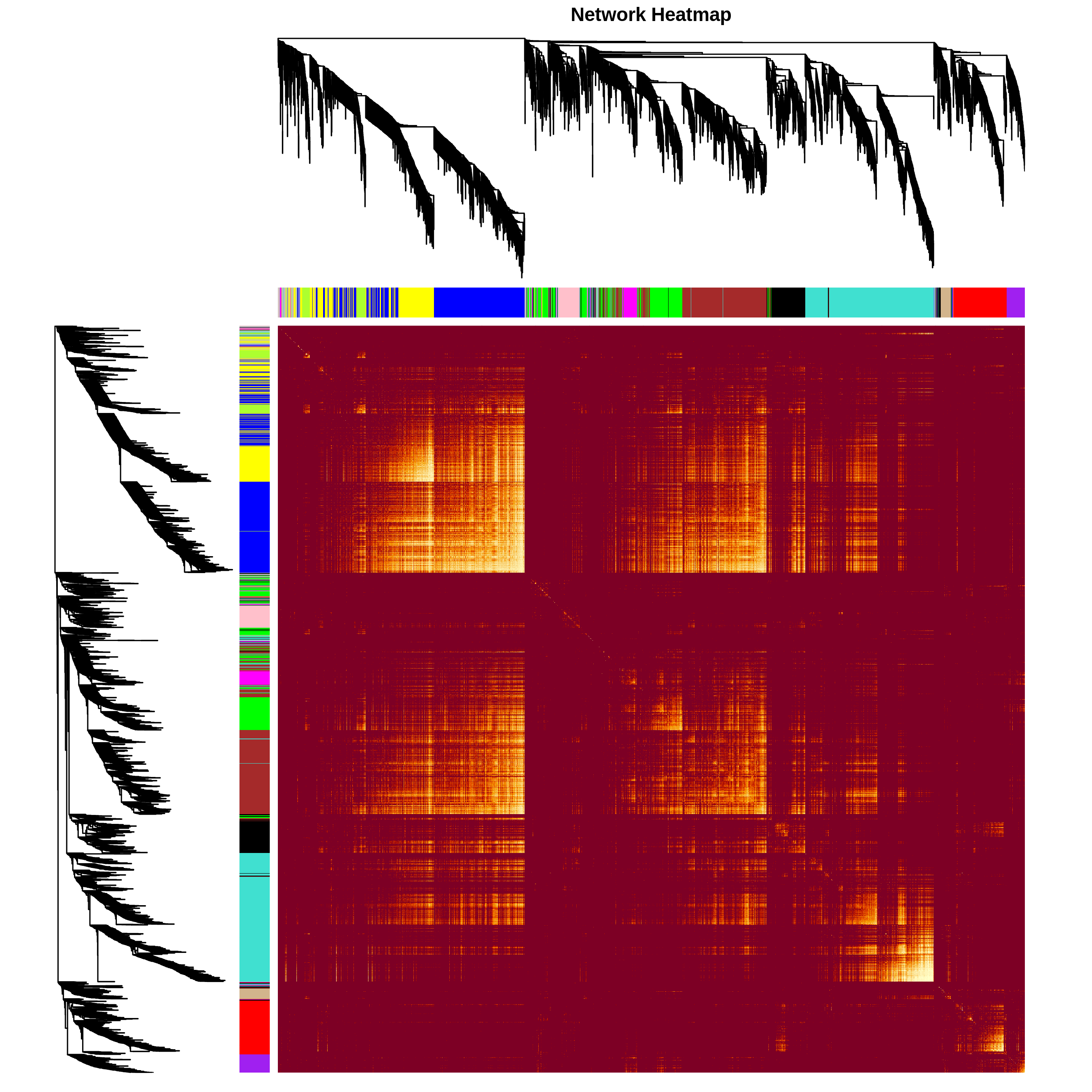

Supplement: Supplementary Figure 4 — The TOM plot with cluster dendrogram. The yellow matrix across diagonal represents the correlation between modules, and the density represents the number of co-expressed genes. [file Image_4.PNG]

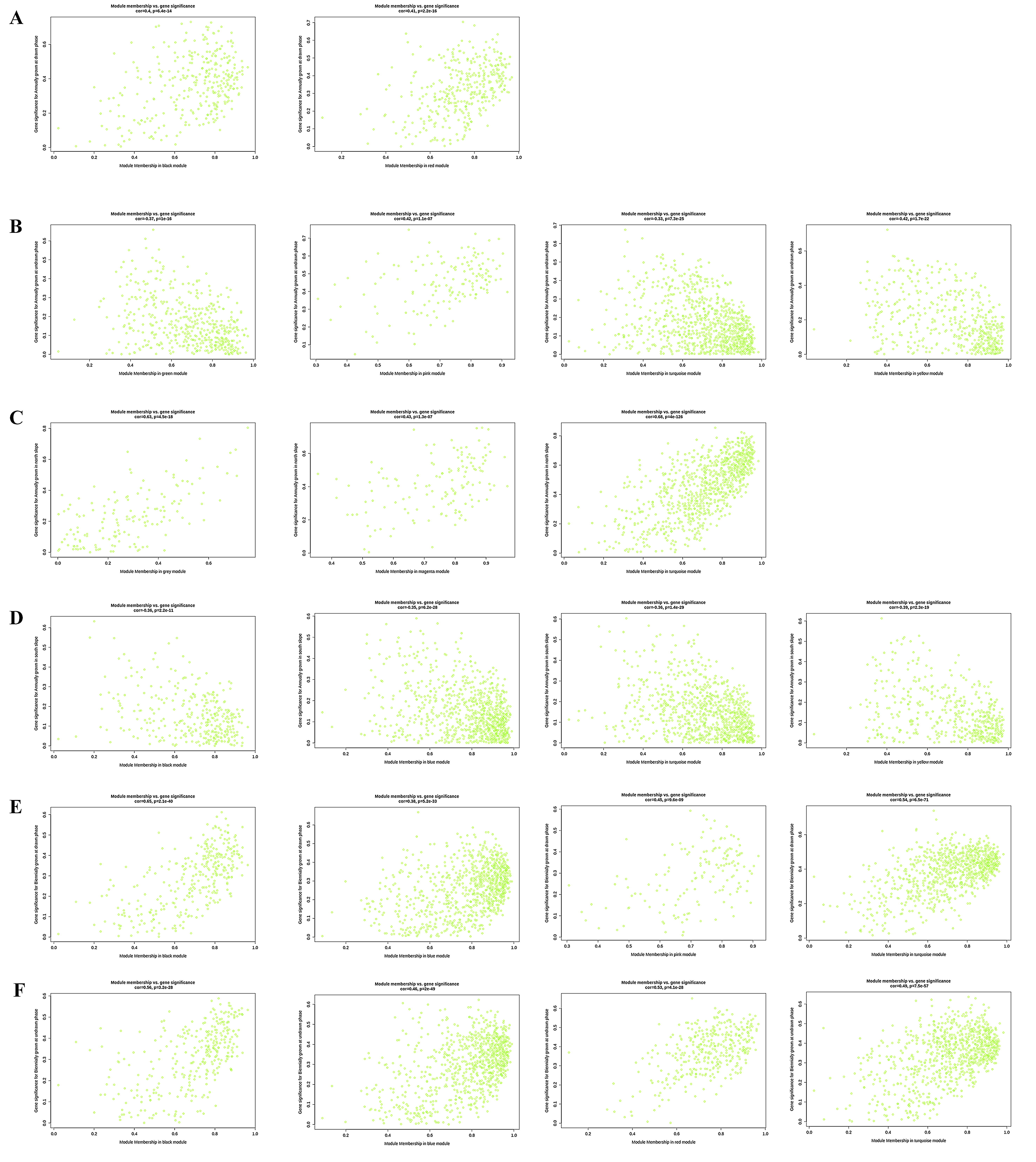

Supplement: Supplementary Figure 5 — The module membership of different conditions with gene significant weight. (A) Annually grown at drawn phase. (B) Annually grown at undrawn phase. (C) Annually grown in north slope. (D) Annually grown in south slope. (E) Biennially grown at drawn phase. (F) Biennially grown at undrawn phase. [file Image_5.JPEG]
