# Supplementary material for: De novo Transcriptome Sequencing Coupled With Co-expression Analysis Reveal the Transcriptional Regulation of Key Genes Involved in the Formation of Active Ingredients in Peucedanum praeruptorum Dunn Under Bolting Period
Source: Front Genet. 2021 Jun 14;12:683037. doi: 10.3389/fgene.2021.683037 (PMC8236723; doi:10.3389/fgene.2021.683037)
Supplement: Supplementary Table 1 — The software version and R packages used in the experiment. [file Data_Sheet_1.docx]

**Table S1** The software version and R packages used in the experiment

| Software Name | Version | R Package | Version |
| --- | --- | --- | --- |
| [Trimmomatic](http://www.usadellab.org/cms/?page=trimmomatic) | 0.36 | vegan | 2.0-10 |
| [FastQC](http://www.bioinformatics.babraham.ac.uk/projects/fastqc/) | 0.11.2 | GO.db | 3.3.0 |
| [NCBI Blast+](https://blast.ncbi.nlm.nih.gov/Blast.cgi) | 2.60 | DEGseq | 1.26.0 |
| [Bowtie2](http://bowtie-bio.sourceforge.net/bowtie2/index.shtml) | 2.3.2 | scatterplot3d | 0.3-36 |
| [SAMtools](http://www.htslib.org/doc/samtools.html) | 1.5 | gplots | 2.17.0 |
| [BCFtools](http://www.htslib.org/doc/bcftools.html) | 1.5 | ballgown | 2.4.3 |
| [Trinity](https://github.com/trinityrnaseq/trinityrnaseq/wiki) | 2.4.0 | topGO | 2.24.0 |
| [HISAT2](https://ccb.jhu.edu/software/hisat2/index.shtml) | 2.1.0 | DESeq2 | 1.12.4 |
| [StringTie](http://www.ccb.jhu.edu/software/stringtie/) | 1.3.3b | ggplot2 | 2.2.1 |
| [GffCompare](https://github.com/gpertea/gffcompare) | 0.10.1 | Hmisc | 4.0-3 |
| [MISA](http://pgrc.ipk-gatersleben.de/misa/) | 1.0 | igraph | 1.0.1 |
| [Primer3](http://primer3.sourceforge.net/) | 2.37 | VennDiagram | 1.6.17 |
| [SnpEff](http://snpeff.sourceforge.net/index.html) | 2.36 | clusterProfiler | 3.0.5 |
| [EricScript](https://sites.google.com/site/bioericscript/) | 0.55 | edgeR | 3.14.0 |
| [BEDTools](http://bedtools.readthedocs.io/en/latest/) | 2.26.0 | WGCNA | 1.51 |
| [KAAS](http://www.genome.jp/tools/kaas/) | 2.1 |  |  |
| [RSeQC](http://rseqc.sourceforge.net/) | 2.6.1 |  |  |
| [Qualimap](http://qualimap.bioinfo.cipf.es/) | 2.2.1 |  |  |
| [Salmon](https://combine-lab.github.io/salmon/) | 0.8.2 |  |  |
| [TransDecoder](http://transdecoder.github.io/) | 3.0.1 |  |  |
| [ASprofile](http://ccb.jhu.edu/software/ASprofile) | 1.0.4 |  |  |
