# Supplementary material for: De novo Transcriptome Sequencing Coupled With Co-expression Analysis Reveal the Transcriptional Regulation of Key Genes Involved in the Formation of Active Ingredients in Peucedanum praeruptorum Dunn Under Bolting Period
Source: Front Genet. 2021 Jun 14;12:683037. doi: 10.3389/fgene.2021.683037 (PMC8236723; doi:10.3389/fgene.2021.683037)
Supplement: Supplementary Table 2 — The detailed information of the samples in the experiment. [file Data_Sheet_2.docx]

**Table S2**  Details of the samples collected in the experiment

| Group | Sample ID | Annotation |
| --- | --- | --- |
| A | A1 | Biennially grown at undrawn phase |
| A | A2 |  |
| A | A3 |  |
| B | B1 | Biennially grown at drawn phase |
| B | B2 |  |
| B | B3 |  |
| C | C1 | Annually grown at undrawn phase |
| C | C2 |  |
| C | C3 |  |
| C | C4 |  |
| C | C5 |  |
| C | C6 |  |
| D | D1 | Annually grown at drawn phase |
| D | D2 |  |
| D | D3 |  |
| D | D4 |  |
| D | D5 |  |
| D | D6 |  |
| E | E1 | Annually grown in north slope |
| E | E2 |  |
| E | E3 |  |
| F | F1 | Annually grown in south slope |
| F | F2 |  |
| F | F3 |  |
