# Supplementary material for: De novo Transcriptome Sequencing Coupled With Co-expression Analysis Reveal the Transcriptional Regulation of Key Genes Involved in the Formation of Active Ingredients in Peucedanum praeruptorum Dunn Under Bolting Period
Source: Front Genet. 2021 Jun 14;12:683037. doi: 10.3389/fgene.2021.683037 (PMC8236723; doi:10.3389/fgene.2021.683037)
Supplement: Supplementary Table 3 — The QC data statistics of all samples. [file Data_Sheet_3.docx]

**Table S3** The QC data statistics of all samples

|  | Total Reads Count(#) | Total Bases Count(bp) | Average Read Length(bp) | Q10 Bases Count(bp) | Q20 Bases Count(bp) | N Bases Count(bp) | N Bases Ratio(%) | GC Bases Count(bp) | GC Bases Ratio(%) |
| --- | --- | --- | --- | --- | --- | --- | --- | --- | --- |
| A1 | 46555670 | 6687915408 | 143.65 | 6687912785 | 6574517874 | 2623 | 0.00% | 2873495105 | 42.97% |
| A3 | 42202122 | 6056528272 | 143.51 | 6056522967 | 5943922212 | 5305 | 0.00% | 2604434268 | 43.00% |
| A2 | 45178572 | 6449000922 | 142.74 | 6448998661 | 6306036273 | 2261 | 0.00% | 2779635351 | 43.10% |
| C3 | 40347362 | 5695088458 | 141.15 | 5695076116 | 5616111427 | 12342 | 0.00% | 2466698939 | 43.31% |
| C2 | 47467746 | 6694520804 | 141.03 | 6694506218 | 6599448399 | 14586 | 0.00% | 2897236330 | 43.28% |
| C1 | 36038308 | 5046203801 | 140.02 | 5046192552 | 4975309973 | 11249 | 0.00% | 2199731617 | 43.59% |
| E1 | 41196046 | 5753604690 | 139.66 | 5753592518 | 5670913373 | 12172 | 0.00% | 2438718471 | 42.39% |
| C6 | 38588096 | 5326822138 | 138.04 | 5326802329 | 5252950720 | 19809 | 0.00% | 2336607687 | 43.86% |
| C5 | 41245326 | 5739437957 | 139.15 | 5739425553 | 5655830212 | 12404 | 0.00% | 2516217026 | 43.84% |
| E2 | 41127014 | 5699872986 | 138.59 | 5699860062 | 5617168592 | 12924 | 0.00% | 2378568763 | 41.73% |
| D5 | 65017940 | 8906680652 | 136.99 | 8906660997 | 8784886776 | 19655 | 0.00% | 3826594401 | 42.96% |
| E3 | 37129868 | 4944777730 | 133.18 | 4944766448 | 4873878971 | 11282 | 0.00% | 2046507629 | 41.39% |
| F1 | 40388966 | 5659044822 | 140.11 | 5659032421 | 5579762457 | 12401 | 0.00% | 2484770357 | 43.91% |
| F2 | 41078548 | 5780387948 | 140.72 | 5780366582 | 5701238385 | 21366 | 0.00% | 2512697904 | 43.47% |
| F3 | 39786866 | 5594915971 | 140.62 | 5594903417 | 5515215702 | 12554 | 0.00% | 2429234843 | 43.42% |
| B1 | 39859106 | 5705110966 | 143.13 | 5705108994 | 5592029721 | 1972 | 0.00% | 2459531033 | 43.11% |
| B2 | 42198140 | 6044989606 | 143.25 | 6044987399 | 5946731183 | 2207 | 0.00% | 2586872809 | 42.79% |
| B3 | 43313612 | 6209552736 | 143.36 | 6209550236 | 6103370074 | 2500 | 0.00% | 2669026917 | 42.98% |
| D6 | 42491368 | 6011782222 | 141.48 | 6011769003 | 5927589595 | 13219 | 0.00% | 2581475436 | 42.94% |
| D4 | 44000044 | 6124388409 | 139.19 | 6124374821 | 6039671067 | 13588 | 0.00% | 2642928283 | 43.15% |
| C4 | 38910154 | 5451947874 | 140.12 | 5451935958 | 5378377656 | 11916 | 0.00% | 2370855639 | 43.49% |
| D2 | 42907202 | 6020956442 | 140.33 | 6020943188 | 5939008494 | 13254 | 0.00% | 2597466550 | 43.14% |
| D3 | 36712846 | 5196859683 | 141.55 | 5196848132 | 5125197191 | 11551 | 0.00% | 2248088798 | 43.26% |
| D1 | 43886060 | 5999882466 | 136.71 | 5999869164 | 5916739890 | 13302 | 0.00% | 2596988549 | 43.28% |
